# Inhibition of Salt‐Inducible Kinase 2 Protects Motor Neurons From Degeneration in ALS by Activating Autophagic Flux and Enhancing mTORC1 Activity

**DOI:** 10.1111/cns.70341

**Published:** 2025-03-26

**Authors:** Weiwei Liang, Chunting Zhang, Di Wang, Xiaoli Su, Xingli Tan, Yueqing Yang, Chaohua Cong, Ying Wang, Di Huo, Hongyong Wang, Shuyu Wang, Xudong Wang, Honglin Feng

**Affiliations:** ^1^ Department of Neurology The First Affiliated Hospital of Harbin Medical University Harbin P.R. China; ^2^ Department of Neurology The Second Affiliated Hospital of Harbin Medical University Harbin P.R. China; ^3^ Division of Life Science and Technology of China, Department of Neurology The First Affiliated Hospital of USTC Hefei P.R. China; ^4^ Department of Neurology Shanghai No. 9 People's Hospital, Shanghai Jiaotong University School of Medicine Shanghai P.R. China

**Keywords:** amyotrophic lateral sclerosis, autophagy, Cu/Zn superoxide dismutase, mTORC1, salt‐inducible kinase 2

## Abstract

**Objectives:**

Autophagic impairment has been implicated in the pathogenesis of amyotrophic lateral sclerosis (ALS). Salt‐inducible kinase 2 (SIK2), a member of the AMP‐activated protein kinase (AMPK) family widely expressed in the central nervous system, plays critical roles in neuronal survival, neurogenesis, and the regulation of autophagy. This study aims to investigate the effects and underlying mechanisms of SIK2 in the pathogenesis of ALS.

**Methods:**

In our work, we used both in vivo and in vitro models of ALS to study the effect of SIK2. Protein and RNA levels were assessed by Western blot, RT‐qPCR, immunofluorescence, and immunohistochemistry. Cell viability and apoptosis were evaluated using CCK‐8 assay and flow cytometry. Transmission electron microscopy was employed to examine autophagic vacuoles. Additionally, lentivirus particles carrying shRNA targeting SIK2 (sh‐SIK2) were injected into the lateral ventricle of ALS mice at 60 days of age. Motor performance was evaluated by the rotarod test.

**Results:**

We observed that increased expression of SIK2 significantly contributed to the degeneration of motor neurons in both the cellular model and the hSOD1^G93A^ transgenic mice model of ALS. SIK2 knockdown enhanced neuronal survival and restored mTORC1 activity. Furthermore, SIK2 suppression facilitated the clearance of mutant SOD1 accumulation by activating autophagic flux and enhancing lysosomal acidification. Conversely, SIK2 overexpression impaired mTORC1 activity, exacerbating autophagy dysfunction by inhibiting lysosomal function, and ultimately led to motor neuron degeneration. In vivo, SIK2 deficiency delayed disease onset and extended the lifespan of ALS mice by enhancing autophagy‐mediated clearance of mutant SOD1 aggregates.

**Conclusions:**

Our findings reveal that SIK2 regulates autophagic flux by modulating lysosomal acidification, thereby influencing the degradation of mutant SOD1 aggregates. SIK2 suppression enhances autophagy‐mediated clearance of toxic protein aggregates and protects motor neurons, highlighting its potential as a therapeutic target for ALS.

## Graphical abstract

Schematic diagram of potential mechanisms by which SIK2 regulates autophagy in ALS. SIK2 contributes to the induction of autophagy by inhibiting mTORC1 signaling. However, SIK2 disrupts lysosomal dysfunction, ultimately resulting in defective autophagy flux. Inhibition of SIK2 promotes mSOD1 accumulation removal by activating autophagy flux and restores mTORC1 activity, thus playing a neuroprotective role on motor neurons in ALS.

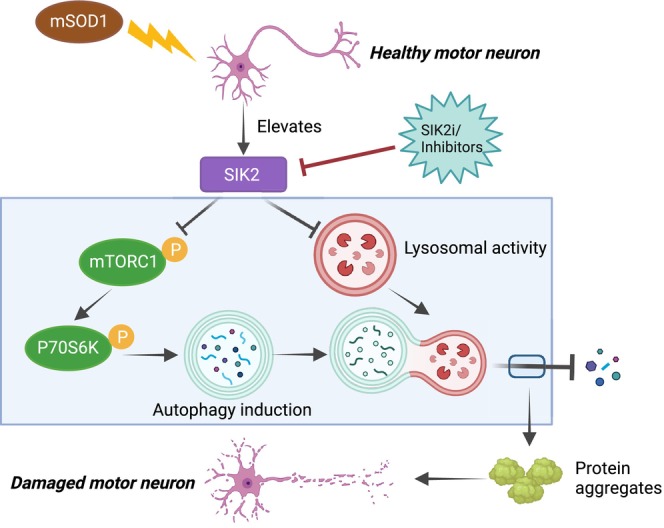

## 1 Introduction

Amyotrophic lateral sclerosis (ALS) is a fatal, adult‐onset neurodegenerative disorder characterized by the progressive loss of upper and lower motor neurons. Familial ALS (fALS) affects about 10% of cases and is attributed to inherited genetic mutations. To date, nearly 30 genes have been implicated in ALS pathogenesis, including chromosome 9 open reading frame 72 (C9orf72), copper/zinc superoxide dismutase (SOD1), transactive response (TAR) DNA‐binding protein 43 (TDP‐43), and fused in sarcoma (FUS) [1]. Among these, pathogenic variants in SOD1 are believed to contribute to 15%–30% of fALS cases and have been extensively studied [2]. Transgenic mice overexpressing human mutant SOD1 provide a widely used model that replicates ALS‐like phenotypes [3], while motor neuron‐like NSC34 cells overexpressing mutant hSOD1 exhibit reduced proliferation and heightened susceptibility to oxidation‐induced cell death, a hallmark of ALS [4]. The toxicity associated with mutant SOD1 is widely regarded as resulting from a gain‐of‐function mechanism rather than a loss‐of‐function [5]. Moreover, recent studies indicate that misfolded wild‐type SOD1 contributes to the pathogenesis of sporadic ALS cases [6]. Although the precise molecular mechanisms underlying toxic protein‐mediated motor neuron degeneration remain unclear, accelerating the clearance of protein aggregates may offer a promising therapeutic approach for ALS treatment.

A crucial pathological hallmark of ALS is the accumulation of protein aggregates and cytoplasmic inclusions in diseased motor neurons [7], indicating impaired protein quality control systems. Unlike most other cell types, post‐mitotic neurons, which do not undergo cell division, are particularly vulnerable to toxic protein species. They require active protein degradation processes to maintain cell homeostasis [8, 9]. Macroautophagy (referred to hereafter as autophagy) is a highly conserved, essential pathway through which cells deliver protein aggregates and damaged organelles to lysosomes for degradation [10]. Multiple studies on post‐mortem tissues from ALS patients have provided evidence of autophagy dysregulation in motor neurons [11, 12, 13]. Furthermore, the accumulation of autophagosomes and endolysosomal deficits observed in the transgenic hSOD1^G93A^ mouse model of ALS suggests a blockage in autophagosomal degradation [14, 15]. Consequently, enhancing autophagic degradation to eliminate toxic protein aggregates is considered a potential therapeutic strategy. The mammalian target of rapamycin (mTOR), a master negative regulator of autophagy, plays a pivotal role in the pathogenesis of neurodegenerative diseases, including ALS [16]. mTORC1 activity has been shown to provide endogenous neuroprotection to motor neurons in fALS [17]. Importantly, lysosomal dysfunction directly influences the activation of the mTORC1 pathway [18, 19]. Whether targeting mTOR is beneficial or harmful depends on the specific disease and the underlying factors contributing to it [20]. Taken together, while autophagy holds significant promise as a therapeutic avenue, its precise role in ALS pathology remains incompletely understood.

Salt‐inducible kinase 2 (SIK2) is a serine/threonine protein kinase belonging to the AMP‐activated protein kinase (AMPK) family, a group of key mediators of energy and stress signaling. SIK2 plays important roles in a variety of biological processes, including the regulation of cancer development and progression [21, 22], neurogenesis [23], neuronal survival [24], and inflammatory responses [25]. Increased SIK2 expression has been observed in neuronal tissues, indicating its significant role in neural physiology [26]. Notably, previous research has demonstrated that SIK2 regulates autophagosome maturation under conditions of proteasome impairment [27]. Additionally, SIK2 has been shown to restrict autophagy flux in triple‐negative breast cancer [28]. Moreover, SIK2 is essential for autophagy in adipocytes through its regulation of TFEB [29] and plays a role in modulating mTORC1–CREB activity in cortical neurons [24]. Collectively, these findings underscore SIK2's specific involvement in autophagic processes. However, the role of SIK2 in the pathogenesis of ALS, as well as the underlying mechanisms, remains unclear.

This study aimed to investigate whether SIK2, a key regulator of autophagy, influences motor neuron survival in ALS. Our findings demonstrate that SIK2 expression is increased in hSOD1^G93A^‐transgenic mice and cellular models of ALS. We further explored the relationship between autophagy alterations and neuronal cell death in vitro. The results indicate that while SIK2 overexpression induces autophagy, increased SIK2 levels impair autophagosome clearance by disturbing lysosomal function, thereby exacerbating motor neuron degeneration. Conversely, silencing SIK2 restores mTORC1 activity and protects motor neurons from misfolded SOD1 toxicity. Additionally, our study reveals a previously unrecognized role of SIK2 in regulating autophagy by inhibiting lysosomal acidification. Notably, hSOD1^G93A^‐transgenic mice injected with LV‐SIK2‐shRNA exhibited a significant delay in disease onset and extended survival, which correlated with enhanced autophagy flux and reduced mutant SOD1 aggregates. Collectively, these findings establish SIK2 as a novel contributor to ALS pathogenesis and highlight its potential as a therapeutic target for neuroprotection through genetic or pharmacological interventions.

## 2 Materials and Methods

### 2.1 Experimental Animals

Transgenic SOD1^G93A^ mice expressing mutant human SOD1 with a Gly93Ala substitution (B6SJL‐Tg (SOD1‐G93A)1Gur/J) [30] were purchased from Jackson Laboratory (Stock no. 002726; Bar Harbor, ME, USA). The mice carrying the mutant SOD1 were genotyped by polymerase chain reaction (PCR) amplification of DNA extracted from tail tissue, as described in our previous reports [31]. Experimental protocols used on animals were approved by the Experimental Animal Research Ethics Committee of Harbin Medical University and performed in accordance with the international guidelines on the ethical use of animals. Efforts were made to minimize the number of animals used and reduce their suffering.

### 2.2 Cell Culture

The mouse neuroblastoma × spinal cord hybrid cell line (NSC34) was a kind gift from Cedarlane Laboratories (University of British Columbia, Canada). This cell line exhibits morphological and physiological properties of motor neurons and has been widely used as a model system for motor neuron studies [32]. The cells were maintained in Dulbecco's modified Eagle's medium (DMEM) supplemented with 10% fetal bovine serum (FBS) and 1% penicillin/streptomycin in a 5% CO_2_ incubator at 37°C.

### 2.3 Production of a Stable hSOD1G93A‐Transfected NSC34 Cell Line

NSC34 cells were cultured and stably transfected with one of the following: (1) an empty puromycin lentivirus vector (PLV‐IRES‐Puro) containing green fluorescent protein (GFP) (pLV cells); (2) a lentivirus vector carrying the wild‐type hSOD1 gene (wtSOD1 cells); or (3) a lentivirus vector carrying the mutant hSOD1‐G93A gene (mSOD1 cells). Stable cell clones were maintained in 200 μg/mL of puromycin (G418, Invitrogen). The relative mRNA levels of the human mSOD1 transgenes were evaluated by quantitative real‐time PCR (RT‐qPCR).

### 2.4 Small‐Interfering RNA Experiment

Cells were transiently transfected with SIK2 siRNA or negative control siRNA (GenePharma, Shanghai, China) using Lipofectamine2000 reagent (Invitrogen, Life Technologies) according to the manufacturer's instructions. At 48 h post‐transfection, the cells were rinsed, and RNA/protein was collected. Three SIK2 siRNA sequences were utilized to evaluate the efficiency of SIK2 mRNA and protein silencing. The sequences used were as follows: (1) SIK2 siRNA‐1: 5′‐GCU AAU CAU GGC CGA UUA ATT‐3′ and 5′‐UUA AUC GGC CAU GAU UAG CTT‐3′. (2) SIK2 siRNA‐2: 5′‐GCC UAG CAC CAU UGC UGA ATT‐3′ and 5′‐UUC AGC AAU GGU GCU AGG CTT‐3′. (3) SIK2 siRNA‐3: 5′‐GGA GUU GAA CAA AGU ACA ATT‐3′ and 5′‐UUG UAC UUU GUU CAA CUC CTT‐3′. For the negative control (si‐NC), the following sequences were used: 5′‐UUC UCC GAA CGU GUC ACG UTT‐3′ and 5′‐ACG UGA CAC GUU CGG AGA ATT‐3′.

### 2.5 Plasmids and Transient Transfections

Flag‐tagged mouse SIK2 expression vector and empty FLAG vector were purchased from GeneCopoeia Inc. (Guangzhou, China). 1 × 10^6^ mSOD1 cells or NSC34 cells were seeded onto 6‐well plates approximately 20 h prior to transfection. Cells were transiently transfected with the Flag‐SIK2 vector or empty FLAG vector using Lipofectamine2000 reagent (Invitrogen, Life Technologies). For the overexpression of SIK2 in mSOD1 cells, the Flag‐SIK2 plasmid (2 μg per well) was diluted in 200 μL Opti‐MEM (Invitrogen, Life Technologies) in separate tubes. Following a 5 min incubation, the Lipofectamine 2000 (4 μL per well) solution was added to the DNA solution to form Lipofectamine 2000/DNA complexes. After a 20 min incubation, the mixture was added dropwise to the cell culture plate. The cells were incubated in 5% CO_2_ for 6 h. After transfection, the cell culture medium was replaced with fresh DMEM containing 2% FBS, and the cells were cultured for 48 h for subsequent experiments.

### 2.6 Immunohistochemistry and Immunofluorescent Staining

Mice were deeply anesthetized with isopentane, and a midline incision was made to expose the chest cavity under sterile conditions. The right atrial appendage was incised for venous drainage, and a sterile needle was inserted into the left ventricle via the cardiac apex. Ice‐cold physiological saline was perfused through the needle until the liver changed from dark red to pale, indicating sufficient vascular flush. The saline was then replaced with 4% paraformaldehyde, and approximately 50 mL was perfused until rigor mortis was observed. Immediately following transcardiac perfusion, the spinal cord and gastrocnemius muscle were rapidly and completely excised. In brief, 8 μm frozen sections of the gastrocnemius muscle were stained with hematoxylin and eosin (H&E). The dissected spinal cord tissues were then fixed, paraffin‐embedded, and sectioned at a thickness of 6 μm, as previously described [33, 34]. The slides were deparaffinized with xylene and hydrated through a series of decreasing alcohol concentrations. Then, the epitopes were exposed with citrate buffer at 96°C for 40 min. Following this, the slides were washed three times with 0.05% Tween20 in PBS and blocked with 5% bovine serum albumin (BSA) for 1 h at room temperature. Slides were then incubated overnight at 4°C with a primary antibody against anti‐SIK2 (1:200, ab53423, Abcam). Subsequently, the slides were incubated with secondary antibodies (Abcam) for 2 h at room temperature. Staining was visualized using 3,3′‐diaminobenzidine (DAB). Images were captured using a Zeiss Axiophot microscope (Carl Zeiss AG, Jena, Germany). Image‐Pro Plus 6.0 software was used to calculate the semiquantitative expression of SIK2. For immunofluorescent staining, sections were incubated with a primary antibody overnight at 4°C. The antibodies used included rabbit anti‐SIK2 (1:200, SAB3500955, Sigma), mouse anti‐MAP‐2 (1:200, microtubule‐associated protein‐2, Beijing Bioss Biotechnology Co), rabbit anti‐Cleaved Caspase‐3 (1:1000, ab49822, Abcam), and mouse anti‐LAMP1 (1:200, 65051‐1‐Ig, proteintech). Secondary antibodies conjugated with Alexa Fluor 488 or 594 (ZSGB‐BIO, Beijing) were applied, and nuclei were counterstained with DAPI (4, 6‐diamidino‐2‐phenylindole, ZSGB‐BIO, Beijing). Images were captured using a fluorescence microscope (Olympus, Tokyo, Japan).

### 2.7 Nissl Staining

Following deparaffinization and hydration, Nissl staining solution was applied to the slides. The slides were then incubated at 37°C for 1 h, followed by two brief washes with double distilled water for 10 s each. Subsequently, the slides were dehydrated through a graded alcohol series and permanently sealed using neutral gelatin. The number of motor neurons (MNs) was counted in three sections per mouse using a Zeiss Axiophot microscope (Carl Zeiss AG, Jena, Germany).

### 2.8 Immunofluorescence Cytochemistry Analysis

Cells were cultured on 24‐well plates, fixed with 4% paraformaldehyde for 15 min at room temperature, permeabilized in PBS containing 0.1% Triton‐100 (Sigma), and subsequently blocked with 5% bovine serum albumin (BSA). The cells were incubated overnight at 4°C with an anti‐SIK2 (1:200, SAB3500955, Sigma) and then with Alexa Fluor 488‐label goat anti‐rabbit IgG (1:200, ZSGB‐BIO). Nuclei were counterstained with DAPI (5 μg/mL, Sigma) for 3 min. Fluorescent images were captured using a fluorescence microscope (Olympus, Tokyo, Japan).

### 2.9 Western Blot Analysis

Cells and spinal cord tissues were homogenized in RIPA buffer (Beyotime Institute of Biotechnology, Jiangsu, China) containing a protease inhibitor (10 μg/mL PMSF). To prevent dephosphorylation, a phosphatase inhibitor (10 mM, Roche, 04693124001) was also applied. Protein concentration was measured using a BCA assay (Beyotime Institute of Biotechnology, China). Western blotting was performed following standard protocols as previously described [33, 34]. The following primary antibodies were used: rabbit anti‐SIK2 (1:1000, #6919, Cell Signaling Technology), mouse anti‐SOD1 (1:500, sc‐271014, Santa Cruz Biotechnology), rabbit anti‐mTOR (1:1000, #2983, Cell Signaling Technology), rabbit anti‐mTOR at serine 2448 (1:1000, #2971S, Cell Signaling Technology), rabbit anti‐P70S6K (1:1000, #9202S, Cell Signaling Technology), anti‐P70S6K at threonine 389 (1:1000, #9202S, Cell Signaling Technology), mouse anti‐SQSTM1/P62 (1:500, ab56416, Abcam), mouse anti‐β‐actin (1:1000, ab8224, Abcam), rabbit anti‐LC3B (1:500, NB100‐2220, Novus), rabbit anti‐Cleaved Caspase‐3 (1:1000, ab49822, Abcam), rabbit anti‐caspase3 (1:2000, ab184787, Abcam), and rabbit anti‐CTSB (1:2000, 12216‐1‐AP, proteintech). Goat anti‐rabbit or anti‐mouse IgG conjugated with Alexa Fluor‐800 secondary antibody (1:10,000, Li‐COR) was used at 37°C for 1 h. Signals were detected using the Odyssey infrared imaging system (Li‐COR Biotechnology, Lincoln, NE, USA) and quantified with Image J software. Signal intensity was normalized to β‐actin, which served as the internal loading control.

### 2.10 Quantitative Real‐Time PCR (RT‐qPCR)

Total RNA was extracted from cells and spinal cord tissues using TRIzol reagent (Invitrogen Life Technologies). cDNAs were synthesized using the FastKing gDNA Dispelling RT SuperMix (TIANGEN BIOTECH, KR118, Beijing). RT‐qPCR for mouse SIK2 and endogenous mouse β‐actin was carried out using the One‐Step SYBR PrimeScript RT‐PCR Kit II (Takara Biotechnology Co., Dalian, China) and the Light Cycler 480 (Roche, Basel, Switzerland) according to the manufacturer's instructions. Each experiment was performed in duplicate, with five independent samples analyzed in triplicate. The following primer sequences were used: SIK2 forward 5′‐TGCCAAGCACCACTTCTTCT‐3′ and reverse 5′‐AAGCAGCTCACAACCCCATT‐3′; β‐actin forward 5′‐CCAGCCTTCCTTGGGTAT‐3′ and reverse 5′‐TGCTGGAAGGTGGACAGTGAG‐3′. The relative mRNA transcription was calculated as a percentage relative to the control conditions using the 2^−ΔΔCT^ method, where Ct is the threshold cycle value.

### 2.11 Cell Apoptosis and Cell Viability Assessments

mSOD1 cells were seeded onto a 6‐well plate and transfected with the SIK2 expression vector or siRNA. After 48 h, the cells were collected and analyzed using flow cytometry with a FACSCalibur (BD Biosciences). For the apoptosis assay, the cells were stained with Annexin V/APC and 7‐AAD (7‐aminoactinomycin) (Biolegend, CA, USA) according to the manufacturer's instructions. Tests were performed in triplicate. Cell viability was assessed using the CCK‐8 assay (Beyotime, CA). Briefly, the cells were plated onto a 96‐well plate and transfected with the SIK2 expression vector or siRNA for 48 h. Subsequently, 10 μL of the CCK‐8 solution was added to each well, and the plate was incubated for 2 h at 37°C. Cell viability was measured by absorbance at 450 nm using a spectrophotometric plate reader (BioTek Instruments, Winooski, VT, USA).

### 2.12 Analysis of SOD1 Aggregation

Detergent‐insoluble mutant SOD1 aggregates were detected by Western blot analysis. NSC34 cells were transiently co‐transfected with human SOD1^G93A^ constructs fused to GFP, along with control siRNA, SIK2 siRNA, FLAG, or SIK2‐FLAG. After 48 h of transfection, total cell extracts were prepared by incubating the cells in 1% Triton X‐100 in PBS containing protease inhibitors for 30 min on ice. The extracts were centrifuged at 3000 rpm for 5 min, and the supernatant was collected and further centrifuged at 13,000 *g* for 20 min to separate Triton X‐100‐soluble and ‐insoluble fractions.

### 2.13 Transmission Electron Microscopy

Cells were fixed with 2.5% glutaraldehyde at 4°C overnight and then post‐fixed for 2 h with fixative B at the same temperature. The samples were dehydrated in a graded acetone series and embedded in EPON 812 resin. After polymerization for 2 days at 70°C, sections were made by an UC6 ultra‐microtome (Leica Microsystems) and stained with uranyl acetate and lead citrate. The samples were analyzed in an H‐7650 transmission electron microscope (Hitachi, Tokyo, Japan) operated at 80 kV.

### 2.14 Lyso‐Tracker Red Staining

Transfected cells were incubated with 50 nM Lyso‐Tracker Red DND‐99 (Beyotime Biotechnology, Shanghai, China) for 1 h at 37°C. Fluorescent images were captured using a fluorescence microscope (Olympus, Tokyo, Japan).

### 2.15 Intracerebroventricular Injection and Behavioral Assessment

The lentivirus carrying SIK2 shRNA (LV‐shSIK2‐mcherry) and the control (LV‐mcherry) were purchased from GenePharma Corporation (Shanghai, China). In the Intracerebroventricular injection model, 10 μL (10^9^ TU/ml) of lentivirus particles was injected into the lateral ventricle of ALS mice at 60 days of age using a glass micro‐needle (Drummond Scientific Company, PA, USA). The injection site was located 1.5 mm lateral, 1.1 mm posterior, and 2.0–2.5 mm deep from the bregma.

Mice were categorized as “pre‐symptomatic” when they exhibited no clinical signs of disease and had not yet attained their peak body weight. The “onset” stage was defined as the point at which mice reached their maximum body weight. The “symptomatic” stage was identified when mice demonstrated a 10% reduction in body weight and exhibited motor impairments, such as tremors or a compromised hindlimb splay reflex. The “late‐symptomatic” stage was characterized by significant hindlimb paralysis, although mice retained the ability to access food and water using their forelimbs. Finally, the “end‐stage” was determined when mice were unable to right themselves within 30 s after being placed in a supine position. Body weight and rotarod performance were measured every 8 days, beginning at 60 days of age, following 1 week of training.

### 2.16 Statistical Analysis

GraphPad Prism 8.0 (GraphPad Software, La Jolla, CA, USA) was used for statistical analysis. Data are presented as mean ± standard deviation (SD) based on at least three independent experiments. Normality was assessed using the Shapiro–Wilk test, and non‐parametric tests were applied where necessary. Student's *t*‐test was used to assess differences between two groups, while a one‐way analysis of variance (ANOVA), followed by Dunnett's post hoc test, was used to evaluate differences among multiple groups. A *p* value < 0.05 was considered statistically significant.

## 3 Results

### 3.1 Increased Expression of SIK2 Was Observed in ALS Pathology

**FIGURE 1:**
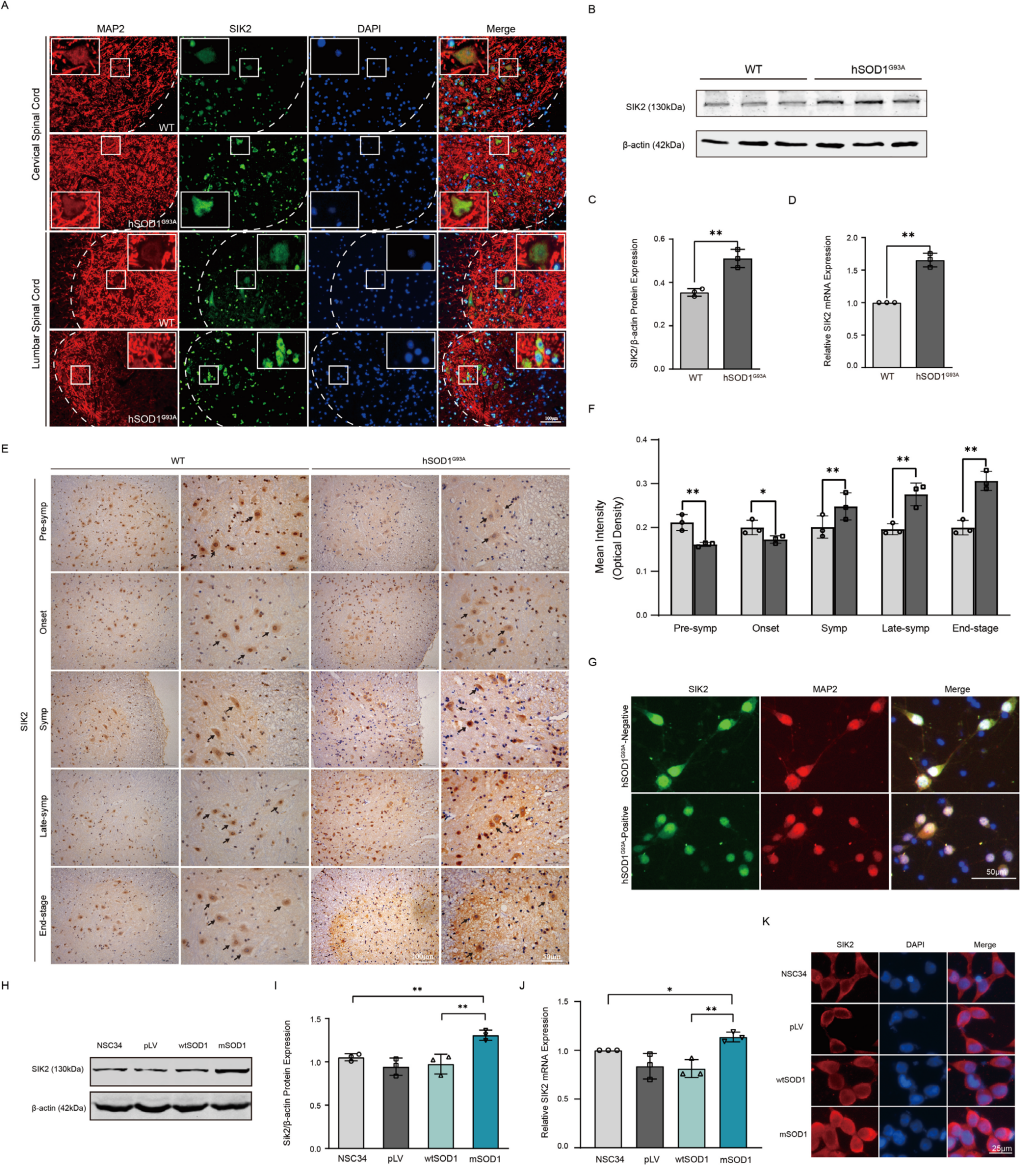
SIK2 levels were changed during ALS pathology. (A) Immunofluorescence of spinal cord sections from 130‐day‐old hSOD1^G93A^‐positive and ‐negative mice using anti‐SIK2 (green) and MAP2 (red) antibodies, with DAPI nuclear stain (blue). SIK2 fluorescence intensity in neurons was higher in hSOD1^G93A^‐positive mice. Scale bar = 25 μm. (B, C) Western blot analysis and quantification of SIK2 protein levels in spinal cord tissues, with β‐actin as a loading control. (D) RT‐qPCR analysis showed that hSOD1^G93A^ transgenic mice showed remarkably increased SIK2 mRNA levels. (E) SIK2‐positive motor neurons (black arrow) were detected in the anterior horn of spinal cords from both hSOD1^G93A^ transgenic and wild‐type mice. Low‐magnification images (left, scale bar = 100 μm); high‐magnification images (right, scale bar = 50 μm). (F) Optical density of SIK2 staining was analyzed using Image‐Pro Plus 6.0 (*n* = 3 per group). (G) Primary cortical neurons from hSOD1^G93A^ embryonic mice were immunostained with anti‐SIK2 (green) and anti‐MAP2 (red), with DAPI nuclear stain (blue). Scale bar = 50 μm. (H, I) Western blot analysis of SIK2 levels in NSC34, pLV, wtSOD1 and mSOD1 cells, with β‐actin as a loading control. Quantification is shown. (J) RT‐qPCR analysis of SIK2 mRNA expression in NSC34, pLV, wtSOD1 and mSOD1 cells, normalized to β‐actin. (K) Representative images of SIK2 immunofluorescence (red) and DAPI (blue) in NSC34, pLV, wtSOD1, and mSOD1 cells. Scale bar = 25 μm. Data represent mean ± SD. Statistical analyses used Student's‐test and one‐way ANOVA with Dunnett's post hoc test. *p < 0.05, **p < 0.01.

To investigate the role of SIK2 in ALS models, we evaluated alterations in SIK2 levels in spinal cord tissues from hSOD1^G93A^‐positive and hSOD1^G93A^‐negative transgenic mice. Immunofluorescence staining for SIK2 was performed on spinal cord sections from 130‐day‐old mice in both groups. SIK2 was detected in both the cytoplasm and nucleus of motor neurons in the ventral horn of the spinal cord. Notably, hSOD1^G93A^‐positive mice exhibited a significant increase in SIK2 fluorescence intensity (green) in motor neurons (red) compared to age‐matched hSOD1^G93A^‐negative mice (Figure 1A). To further validate these findings, Western blot analysis was conducted to semi‐quantify SIK2 protein levels in the spinal cord. Consistent with the immunofluorescence results, hSOD1^G93A^‐positive mice showed significantly increased SIK2 protein levels compared to hSOD1^G93A^‐negative controls (Figure 1B,C). The induced SIK2 mRNA expression correlated with increased SIK2 protein levels (Figure 1D). To systematically evaluate SIK2 expression patterns in ALS, we analyzed SIK2 levels across different disease stages [35, 36]: the pre‐symptomatic stage (75 days), disease onset (95 days), symptomatic stage (120 days), late‐symptomatic stage (130 days), and end‐stage. Interestingly, we observed a biphasic pattern in SIK2 protein expression, with an initial decrease followed by an increase as the disease progressed (Figure 1E,F). Furthermore, SIK2 expression levels were examined in primary cultured neurons. SIK2 staining was slightly reduced in mSOD1‐positive neurons compared to mSOD1‐negative neurons (Figure 1G).

Next, we investigated SIK2 levels in cellular models of ALS. NSC34 cells stably transfected with a lentiviral vector containing hSOD1^G93A^ (mSOD1 cells) were used as a classic ALS disease model [37]. These cells exhibit key pathological characteristics of ALS, including mitochondrial dysregulation [38], Golgi apparatus fragmentation [39], and reduced viability [40]. Western blot analysis revealed significantly increased SIK2 protein expression in mSOD1 cells compared to NSC34, pLV, and wtSOD1 cells (Figure 1H,I). Similarly, RT‐qPCR analysis demonstrated elevated SIK2 mRNA levels in mSOD1 cells relative to the control groups (Figure 1J). Furthermore, immunofluorescence showed robustly enhanced SIK2 immunoreactivity in mSOD1 cells, whereas NSC34, pLV, and wtSOD1 cells exhibited comparable fluorescence intensities (Figure 1K). These findings indicate that SIK2 expression is markedly upregulated in spinal cord motor neurons at the symptomatic and late‐symptomatic stages of hSOD1^G93A^ mice and in the cellular ALS model. Thus, we hypothesize that SIK2 plays an essential role in the pathophysiological processes of ALS.

### 3.2 Autophagy Flux Was Impaired in SOD1^G93A^ ‐NSC34 Cells

**FIGURE 2:**
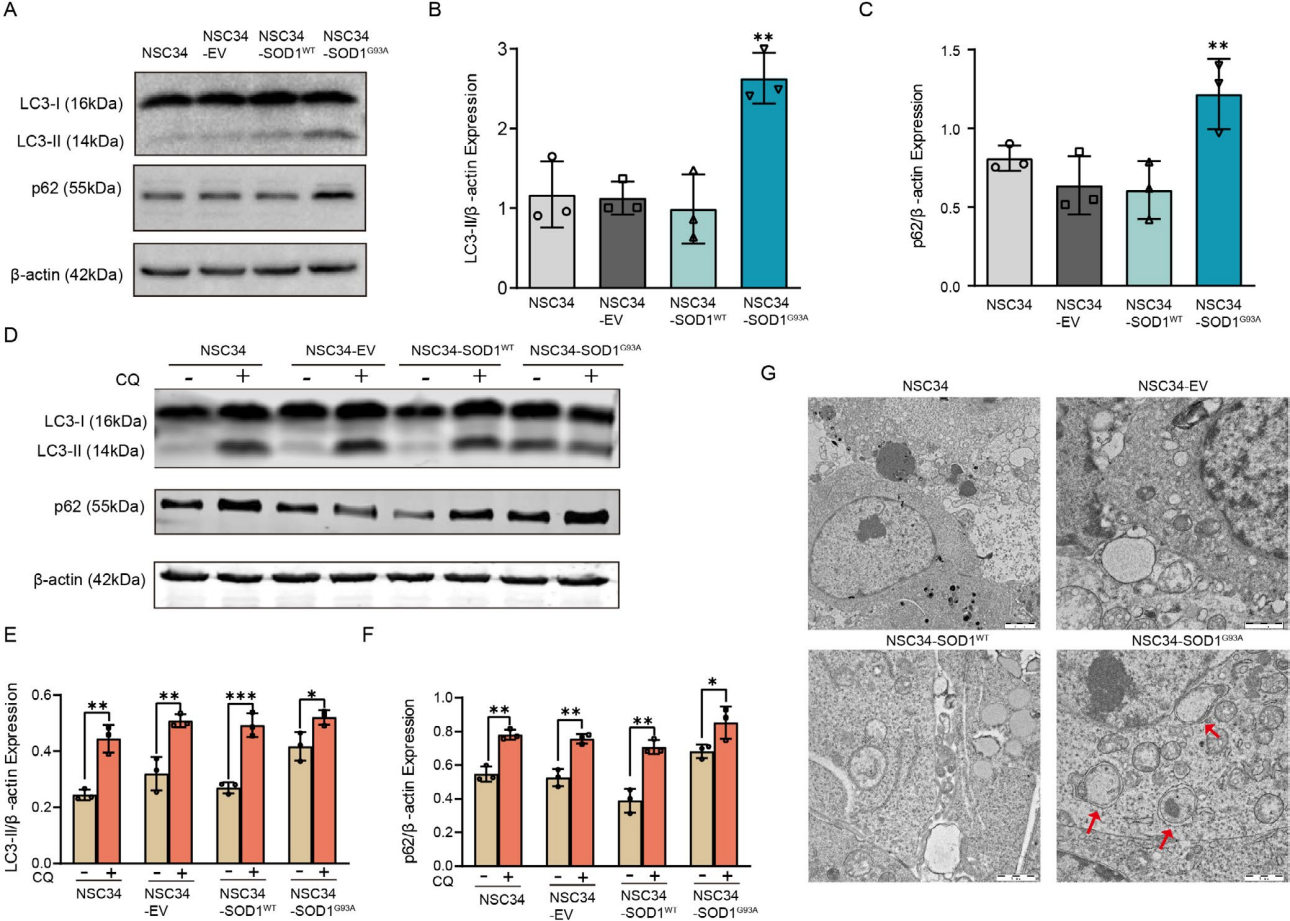
Impaired autophagic flux in SOD1^G93A^‐NSC34 cells. (A) Western blot analyses of LC3 and p62 protein levels in NSC34 cells from four groups:NSC34, NSC34‐EV, NSC34‐SOD1^WT^, and NSC34‐SOD1^G93A^. β‐actin was used as a loading control. (B, C) Quantifications of LC3‐II and p62 levels normalized to β‐actin. (D) NSC34, NSC34‐EV, NSC34‐SOD1^WT^, and NSC34‐SOD1^G93A^ cells were treated with 50 μM chloroquine (CQ) for 4 h. Western blot analysis of LC3 and p62 levels was conducted, with β‐actin as a loading control. (E, F) Quantifications of LC3‐II and p62 levels normalized to β‐actin. (G) Electron microscopy showed that double‐membrane autophagosomes were present in the cytoplasm of SOD1^G93A^‐NSC34 cells (Scale bar = 1 μm). Data represent mean ± SD (*n* = 3). One‐way ANOVA and Dunnett's post hoc test were used to evaluate statistical significance, *p < 0.05, **p < 0.01, ***p < 0.001.

To monitor LC3 levels in cells expressing mutant SOD1, we transiently transfected motoneuron NSC34 cells with an empty vector (EV), human wild‐type SOD1 (SOD1^WT^), or human mutant SOD1‐G93A (SOD1^G93A^). LC3‐I converts to LC3‐II and localizes to phagophores during autophagosome formation [41]. After 72 h of transfection, the expression of SOD1^G93A^ significantly increased LC3‐II levels, while EV‐ and SOD1^WT^‐transfected cells exhibited comparable LC3‐II protein levels (Figure 2A,B). However, we could not confirm whether the elevation in LC3‐II levels resulted from autophagy induction or impaired autophagosome turnover. To clarify the involvement of autophagy, we analyzed p62 protein expression. p62 is an autophagy adaptor that binds to LC3‐II and is degraded through autophagy [42]. Decreased p62 levels typically indicate increased autophagic flux, making it a reliable marker for assessing autophagy [43]. Western blot analysis revealed significantly higher p62 levels in SOD1^G93A^–overexpressing cells compared to control groups (Figure 2A,C). These findings suggest that the autophagy pathway may be disrupted in SOD1^G93A^–overexpressing cells. To further assess autophagic flux, transfected cells were treated with chloroquine (CQ), a lysosomotropic agent that inhibits lysosomal acidification. CQ treatment resulted in significantly increased LC3‐II and p62 levels in NSC34, EV‐NSC34, and SOD1^WT^‐NSC34 cells compared to SOD1^G93A^‐NSC34 cells (Figure 2D–F), indicating partial impairment of autophagy flux due to SOD1^G93A^ overexpression. Additionally, transmission electron microscopy revealed a marked increase in autophagic vacuoles in SOD1^G93A^‐NSC34 cells compared with control groups (Figure 2G). Collectively, these results indicate that mutant SOD1^G93A^ disrupts autophagic flux, leading to autophagosome accumulation.

### 3.3 Suppression of SIK2 Protected Cells Against Misfolded SOD1‐Induced Toxicity

**FIGURE 3:**
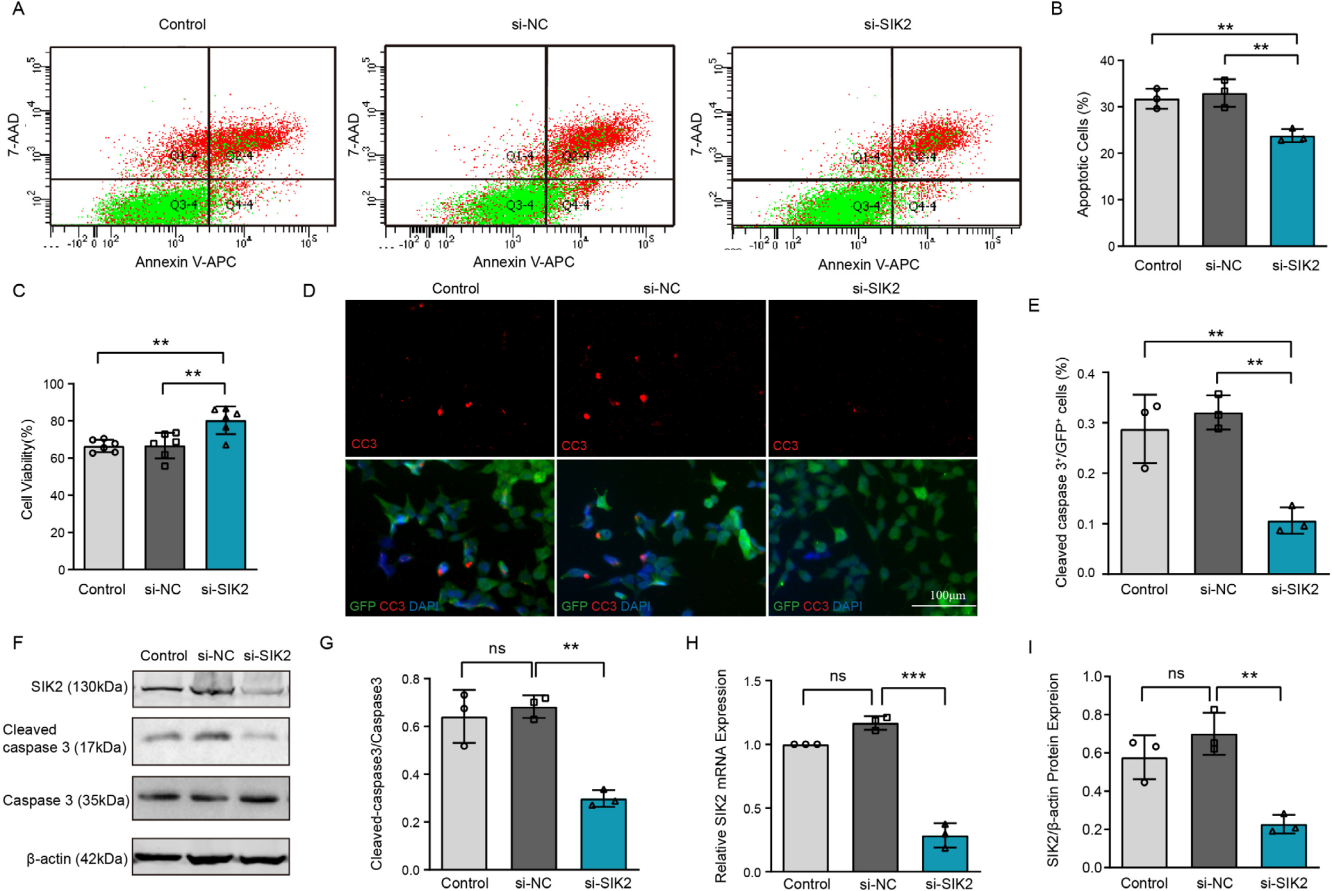
Down‐regulation of SIK2 improves motor neurons survival. (A) Cell apoptosis was assessed by flow cytometric following SIK2 silencing. Cells were co‐stained with Annexin V‐APC and 7‐AAD. Annexin V‐APC stained cells (Q4‐4) = early apoptotic cells; Annexin V‐APC/7‐AAD stained cells (Q2‐4) = cells at the later stage of apoptosis. Apoptosis incidence = Q2‐4 + Q4‐4. (B) Quantifications of (A). (C) Cell viability was quantified using the CCK8 assay. (D, E) MSOD1 cells transfected with control siRNA or SIK2 siRNA were stained with anti‐cleaved caspase3 (CC3, red) and quantified. DAPI was used to counterstain nuclei (Scale bar = 100 μm). More than 100 cells were quantified per replicate experiment. (F, G and I) Cleaved caspase3 and SIK2 protein levels were analyzed by Western blot and quantified. β‐actin was used as a loading control. (H) SIK2 mRNA levels were determined using RT‐qPCR. Data are presented as mean ± SD (*n* = 3). One‐way ANOVA and Dunnett's post hoc test were used to evaluate statistical significance, **p < 0.01, ***p < 0.001, ns: not significant.

To investigate the consequences of increased SIK2 levels in ALS pathogenesis, we assessed the impact of SIK2 repression on mutant SOD1‐induced toxicity. Flow cytometry was used to measure apoptosis levels, revealing that silencing SIK2 reduced the percentage of apoptotic cells (Figure 3A,B). Additionally, cell viability was evaluated using the CCK8 assay, which showed that SIK2 knockdown partially restored survival in cells expressing the G93A mutation (Figure 3C). Moreover, we monitored cleaved caspase‐3 levels through immunostaining to examine the effect of SIK2 inhibition on neuronal death. SIK2 depletion reduced the number of cleaved caspase‐3‐positive cells (Figure 3D,E). Consistent with these results, Western blot analysis showed a significant reduction in cleaved caspase‐3 protein levels in mSOD1 cells transfected with SIK2‐targeting siRNA (Figure 3F,G). The efficiency of SIK2 knockdown was confirmed using qRT‐PCR and Western blotting (Figure 3H,I). Together, these results suggest that SIK2 exerts a cytoprotective role by mitigating misfolded SOD1‐induced toxicity.

**FIGURE 4:**
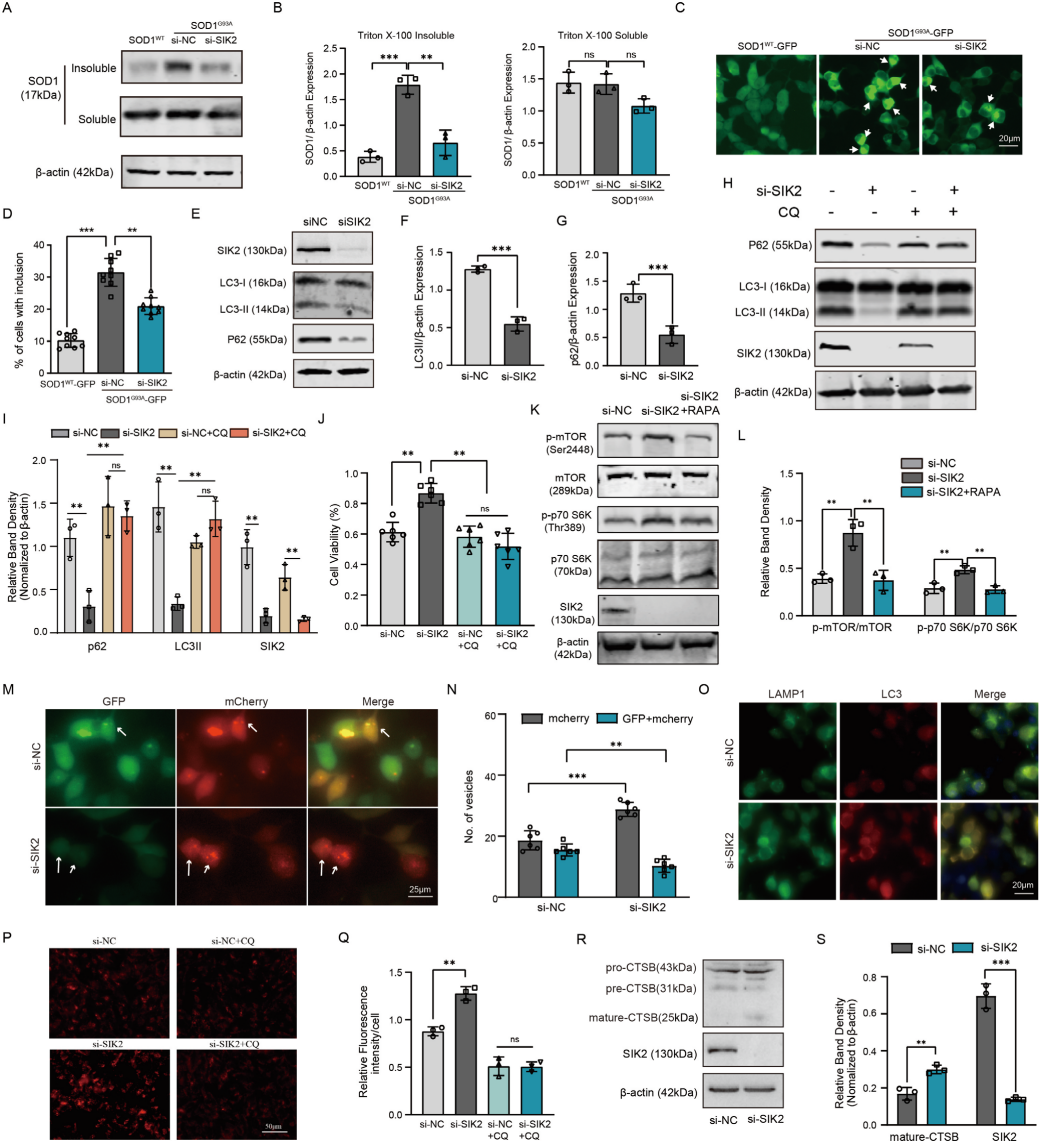
SIK2 deficiency reduces mutant SOD1 through activating autophagic flux and increased mTORC1 activity. (A) SOD1 aggregates were analyzed in Triton X‐100‐insoluble and ‐soluble fractions by Western blot. (B) Quantifications of SOD1 aggregates from (A). (C) SOD1 intracellular inclusions were visualized by fluorescent microscopy (arrows). (D) Percentage of cells with inclusions was quantified in at least 100 cells per replicate. (E–G) LC3‐II and p62 levels were assessed by Western blot and quantified in SIK2 knockdown mSOD1 cells. β‐actin was used as a loading control. (H, I) Autophagic flux was evaluated in SIK2‐deficient mSOD1 cells treated with CQ for 4 h. LC3‐II and p62 levels were quantified. (J) Cell viability was measured using the CCK8 assay. (K, L) mTOR pathway proteins were analyzed by Western blot in SIK2 knockdown mSOD1 cells. β‐actin was used for normalization. (M, N) Immunofluorescence using mCherry‐GFP‐LC3B lentiviral vector (scale bar: 25 μm). GFP (autophagosomes) and mCherry (autolysosomes) puncta were quantified. (O) Immunofluorescence showing LC3B and LAMP1 colocalization in si‐SIK2 and si‐NC groups (scale bar: 20 μm). (P, Q) Lyso‐Tracker Red dye fluorescence in mSOD1 cells of different groups (Scale bar = 50 μm). Relative fluorescence intensity was quantified. (R, S) Western blot analysis of CTSB and SIK2 in si‐NC and si‐SIK2 groups, with band density quantification. Data represent mean ± SD (*n* = 3). One‐way ANOVA and Dunnett's post hoc test were used to evaluate statistical significance, **p < 0.01, ***p < 0.001, ns: not significant.

Since SOD1 mutations lead to the formation of SOD1 aggregates and subsequent motor neuron death [44], we hypothesized that SIK2 inhibition might enhance motor neuron viability partly by reducing mutant SOD1 accumulation. To test this hypothesis, NSC34 cells with depleted SIK2 were transiently co‐transfected with expression vectors for human SOD1^WT^ or human mutant SOD1^G93A^ as GFP fusion proteins. Western blot analysis revealed a significant reduction in Triton X‐100‐insoluble mutant SOD1 levels in SIK2 siRNA‐treated NSC34 cells compared to the control groups. Although a decrease in Triton X‐100‐soluble mutant SOD1 levels was observed in the si‐SIK2 group compared to the si‐NC group, this difference was not statistically significant (Figure 4A,B). Furthermore, fluorescence microscopy of intracellular SOD1 inclusions confirmed these findings, showing that SIK2 suppression reduced the number of inclusions formed by SOD1^G93A^ (Figure 4C,D). Collectively, these results indicate that SIK2 knockdown facilitates the clearance of SOD1 aggregates and promotes motor neuron survival.

### 3.4 SIK2 Deficiency Alleviates Defective Autophagic Flux and Regulates mTORC1 Activity

Previous studies have shown that SIK2 is involved in regulating autophagy [28, 45], a critical pathway for the effective removal of aggregated proteins [46, 47]. We speculated that the decrease in mutant SOD1 aggregation observed with SIK2 down‐regulation could be due to the activation of the autophagy‐lysosome pathway. To test this, we evaluated LC3‐II and p62 levels in mSOD1 cells treated with control siRNA or SIK2 siRNA. After 48 h of transfection, SIK2 siRNA obviously reduced LC3‐II and p62 levels (Figure 4E–G). To further investigate whether this effect was due to autophagy activation induced by SIK2 inhibition, we measured autophagic flux by exposing both SIK2 siRNA and control siRNA to CQ. After 4 h of CQ treatment, SIK2 siRNA increased LC3‐II and p62 levels in both siRNA‐treated groups, indicating that SIK2 knockdown induces autophagic flux (Figure 4H,I). In addition, CQ abolished the neuroprotective effect of SIK2 suppression in mSOD1 cells, as cell viability significantly decreased in the si‐NC + CQ and si‐SIK2 + CQ groups (Figure 4J). Together, our data show that SIK2 deficiency promotes autophagic flux, contributing to the degradation of mutant SOD1.

Previous research has also revealed that SIK2 interacts with mTORC1, a major regulator of autophagy [22, 48]. We hypothesized that SIK2 may regulate mTOR signaling to induce autophagy. Western blot analysis of SIK2, mTOR, p‐mTOR, p70S6K, and p‐p70S6K revealed that SIK2 knockdown increased phosphorylation of mTOR and p70S6K, indicating mTORC1 activation (Figure 4K,L). Inhibition of mTOR signaling with rapamycin (RAPA) reduced p‐mTOR and p‐p70S6K levels in the si‐SIK2 + RAPA group compared to the SIK2 knockdown group (Figure 4K,L). These results suggest that SIK2 inhibition enhances autophagic degradation of mutant SOD1 and activates mTORC1. Phosphorylation of mTORC1 is known to inhibit autophagosome formation [49], but SIK2 inhibition activates autophagic flux, suggesting that SIK2 plays a role in autophagic lysosomal degradation. To validate this hypothesis, we used a tandem‐labeled pCMV‐mCherry‐GFP‐LC3B reporter to measure autophagic flux. GFP, a stably folded protein resistant to lysosomal proteases, loses its fluorescence signal in the acidic environment of lysosomes. In this system, autophagosomes are labeled yellow (mCherry and GFP), while autolysosomes are labeled red (mCherry only). SIK2 suppression caused a prominent increase in red dots, indicating an overall increase in acidic autolysosomes, while yellow dots decreased in the SIK2 inhibition group compared to controls (Figure 4M,N). These data demonstrate that SIK2 knockdown enhances autophagic vesicle acidification.

Autophagic degradation relies on the fusion of autophagosomes with lysosomes and the functional integrity of lysosomes [50]. We examined the localization of LC3B and the lysosomal marker LAMP1, observing colocalization in both SIK2 knockdown and control groups (Figure 4O). To assess whether SIK2 influences lysosomal function, we used the LysoTracker Red probe, a weakly alkaline fluorescent marker that accumulates in acidic lysosomes [51]. SIK2 suppression significantly increased LysoTracker Red fluorescence intensity compared to controls. As expected, CQ treatment reduced fluorescence intensity in SIK2‐down‐regulated groups (Figure 4P,Q). Furthermore, SIK2 suppression strongly increased the mature forms of the lysosomal hydrolase cathepsin B, a key lysosomal hydrolase extensively studied for its role in lysosomal degradation [52] (Figure 4R,S). Collectively, these findings provide compelling evidence that SIK2 down‐regulation promotes autophagic flux by enhancing lysosomal acidification.

### 3.5 Overexpression of SIK2 Aggravated Autophagy Flux, Impaired mTORC1 Activity, and Triggered Neurodegeneration

**FIGURE 5:**
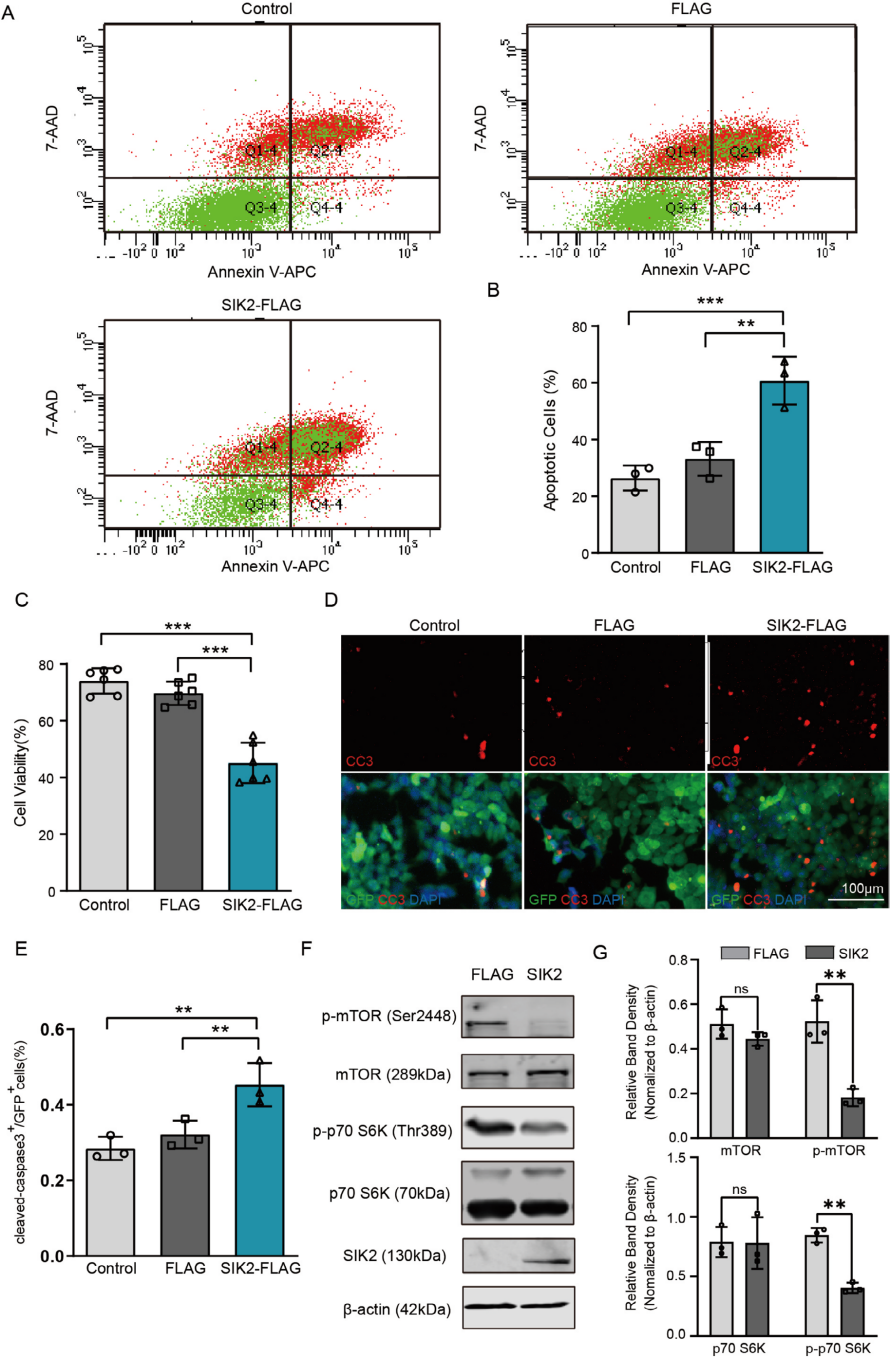
Overexpression of SIK2 triggers neuronal cell apoptosis. (A) Flow cytometric was used to detect apoptosis in mSOD1 cells overexpression SIK2, using Annexin V‐APC and 7‐AAD assays. Annexin V‐APC stained cells (Q4‐4) = early apoptotic cells; Annexin V‐APC/7‐AAD stained cells (Q2‐4) = cells at a later stage of apoptosis. Apoptosis incidence = Q2‐4 + Q4‐4. (B) Quantification of apoptosis cells from (A). (C) Cell viability was assessed using CCK8 assays. (D) Representative images of anti‐cleaved caspase‐3 (green) and DAPI (blue) fluorescence staining. (Scale bar = 100 μm). (E) Quantification of cleaved caspase‐3‐positive cells from (D). More than 100 cells were quantified per replicate. (F, G) Western blot analysis and quantification of SIK2, LC3, p62, and Beclin1 levels. β‐actin was used as a loading control. Data represent mean ± SD (*n* = 3). One‐way ANOVA and Dunnett's post hoc test were used to evaluate statistical significance, **p < 0.01, ***p < 0.001, ns: not significant.

In reciprocal experiments, we investigated the effect of SIK2 upregulation on cell survival in mSOD1 cells. mSOD1 cells were transiently transfected with either Flag‐SIK2 or control Flag plasmids, and apoptosis levels were measured at 48 h post‐transfection using flow cytometry. Exogenous expression of SIK2 significantly increased apoptosis, with mSOD1 cells in the Flag‐SIK2 group exhibiting the highest percentage of apoptotic cells (Figure 5A,B). Consistently, the CCK8 assay revealed that SIK2 overexpression reduced cell viability (Figure 5C). Furthermore, SIK2 overexpression markedly increased the number of cleaved caspase‐3‐positive cells (Figure 5D,E). To determine whether SIK2 overexpression affects mTOR signaling, we examined the phosphorylation status of mTOR and its downstream target, p70S6K. Western blot analysis showed that the levels of p‐mTOR and p‐p70S6K were significantly reduced in the Flag‐SIK2 group compared to the control group (Figure 5F,G).

**FIGURE 6:**
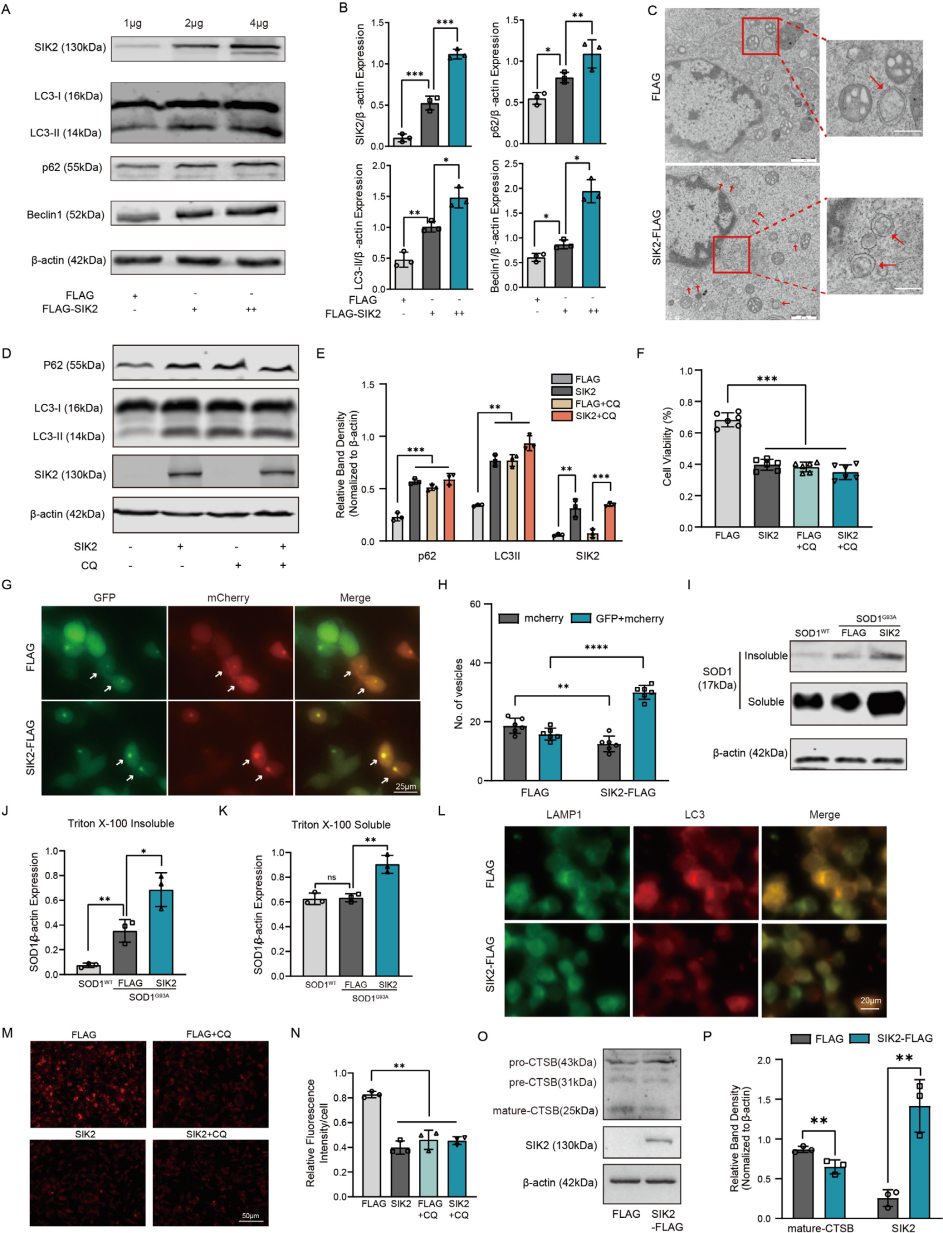
SIK2 impairs mTORC1 activity and inhibits autophagic degradation of mutant SOD1. (A and B) Western blot analysis of LC3, P62, and Beclin1 levels in mSOD1 cells transfected with increasing concentrations of SIK2‐FLAG (1, 2, and 4 μg) for 24 h. Quantification is shown. (C) Electron microscopy of mSOD1 cells transfected with SIK2‐FLAG or FLAG vectors, showing autophagosomes (red arrows, scale bar = 1 μm). (D, E) Whole‐cell extracts in FLAG, SIK2‐FLAG, CQ and SIK2‐FLAG plus CQ were subjected to western blots. Quantifications of LC3‐II and p62 levels in normalized to β‐Actin. (F) Cell viability was assessed using CCK8 assay in mSOD1 cells transfected with FLAG or SIK2‐FLAG and treated with CQ. (G, H) Immunofluorescence using the pCMV‐mCherry‐GFP‐LC3B vector to quantify autophagosomes (yellow) and autolysosomes (red) in FLAG and SIK2‐FLAG groups. (Scale bar: 25 μm). (I–K) Mutant SOD1 aggregation were evaluated by Western blot and quantified. (L) Immunofluorescence showing LC3B and LAMP1 colocalization in FLAG and SIK2‐FLAG groups (scale bar: 20 μm). (M, N) Lyso‐Tracker Red fluorescence in mSOD1 cells from different groups (Scale bar = 50 μm) and quantification of fluorescence intensity. (O, P) Western blot analyses and quantification of CTSB and SIK2 in FLAG and SIK2‐FLAG groups. Data represent mean ± SD (*n* = 3). One‐way ANOVA and Dunnett's post hoc test were used to evaluate statistical significance, *p < 0.05, **p < 0.01, ***p < 0.001, ****p < 0.0001, ns: not significant.

To evaluate the effect of SIK2 overexpression on the autophagy pathway, we measured the levels of key autophagy markers. As expected, ectopic expression of SIK2 significantly increased LC3‐II, Beclin1, and p62 levels in a dose‐dependent manner, indicating enhanced autophagy induction (Figure 6A,B). Consistently, transmission electron microscopy revealed a notable increase in autophagosomes in mSOD1 cells transfected with SIK2‐FLAG vectors (Figure 6C). To determine whether these findings reflect a blockage of autophagy, autophagic flux was assessed in mSOD1 cells overexpressing SIK2‐FLAG or FLAG vectors in the presence or absence of CQ. In the SIK2 overexpression group, CQ treatment did not cause a further increase in LC3‐II levels, suggesting that SIK2 does not directly influence autophagosome biogenesis. Similarly, no significant differences in p62 levels were observed between the SIK2‐FLAG group with or without CQ treatment (Figure 6D,E). Additionally, SIK2 overexpression did not alter cell viability in the presence of CQ (Figure 6F). The number of non‐acidic puncta (GFP + mCherry) was markedly increased in the SIK2 overexpression group, while the number of acidic puncta (mCherry) decreased (Figure 6G,H). To further explore the impact of SIK2 on mutant SOD1 aggregation, we analyzed Triton X‐100‐insoluble and soluble mutant SOD1 levels in SOD1^G93A^‐NSC34 cells. Overexpression of SIK2 significantly increased both forms of mutant SOD1 (Figure 6I–K), suggesting that lysosomal degradation may be compromised by SIK2 upregulation. Interestingly, SIK2 overexpression did not affect the colocalization of LC3B and LAMP1, indicating that SIK2 does not impair autophagosome –lysosome fusion (Figure 6L). Notably, Lyso‐Tracker Red fluorescence intensity was weaker in SIK2‐overexpression mSOD1 cells compared to controls, and CQ treatment reduced Lyso‐Tracker staining similarly to that observed in the SIK2 upregulated group (Figure 6M,N). Furthermore, SIK2 overexpression inhibited both the expression and enzymatic activity of cathepsin B (Figure 6O,P). These findings suggest that SIK2 overexpression promotes mutant SOD1 aggregation and reduces lysosomal acidification, implicating SIK2 in the regulation of lysosomal degradation. Moreover, the compromised autophagic flux and impaired mTORC1 activity caused by SIK2 ultimately lead to neuronal demise.

### 3.6 Neuroprotective Effects of Intracerebroventricular Injection of LV‐shSIK2 in ALS Mice

**FIGURE 7:**
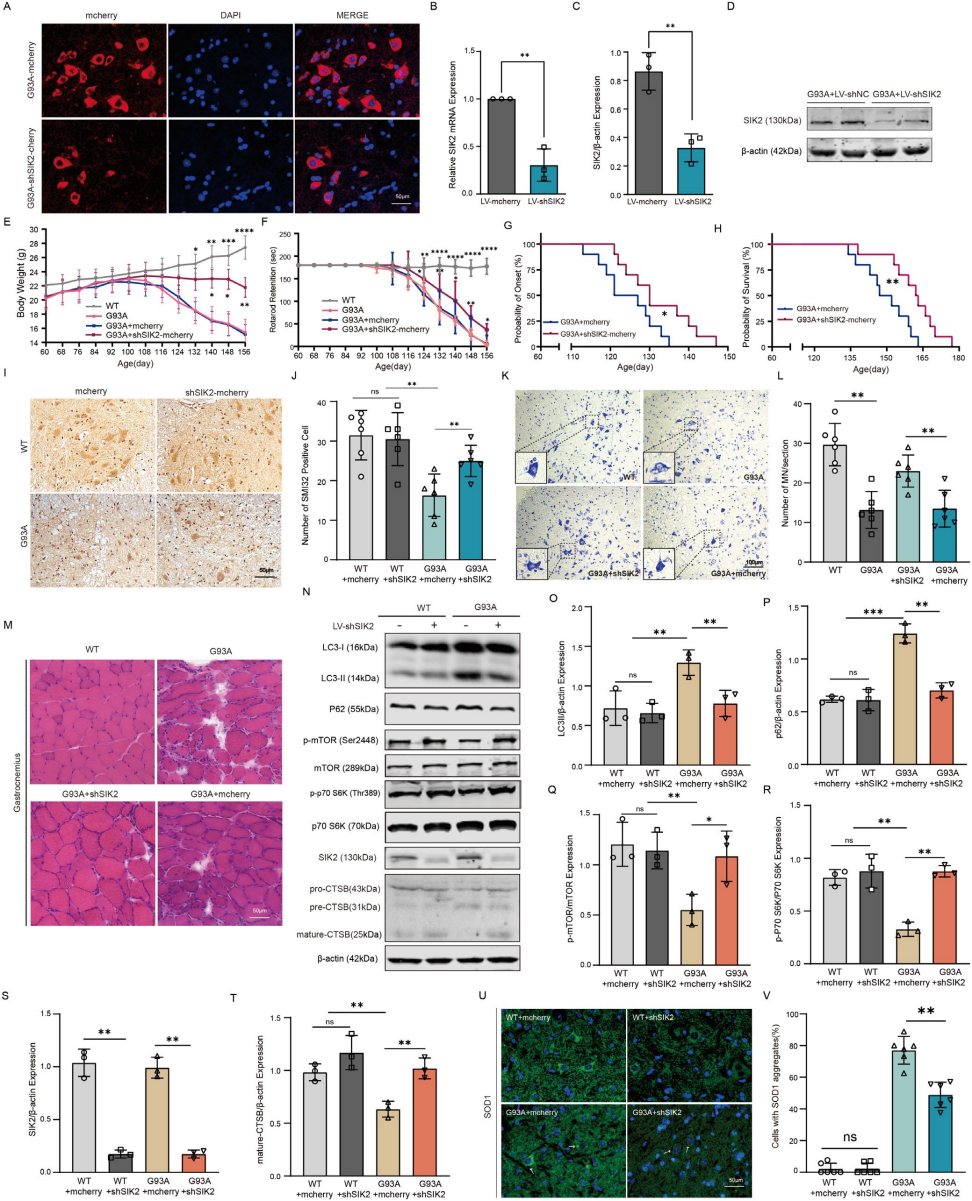
Down‐regulation of SIK2 plays a protective role in ALS mice by promoting autophagic flux. (A) Representative fluorescent images of the spinal cord in ALS mice injected with LV‐mCherry or LV‐shSIK2‐mCherry, verified by mCherry autofluorescence. (Scale bar = 50 μm) (B) SIK2 mRNA levels in the spinal cords of ALS mice injected with LV‐mCherry or LV‐shSIK2‐mCherry, measured by RT‐qPCR. (C, D) SIK2 protein levels in the spinal cords of ALS mice injected with LV‐mCherry or LV‐shSIK2‐mCherry, measured by Western blot and quantified. (E, F) Body weight and motor function were recorded in both groups. (G, H) Disease onset and survival time were analyzed using Kaplan–Meier survival analysis. (I) Immunohistochemical staining of SMI32 in the anterior horn of spinal cords from end‐stage ALS mice. (Scale bar = 50 μm) (J) Quantification of SMI32‐positive motor neurons. (K, L) Nissl staining was performed, and the number of Nissl bodies in each section was quantified. (Scale bar = 100 μm). (M) Hematoxylin and eosin (H&E) staining of gastrocnemius muscle. (Scale bar = 50 μm) (N–T) Western blot analysis and quantification of LC3II, P62, p‐mTOR, mTOR, p‐p70 S6K, p70 S6K, mature‐CTSB, and SIK2 protein levels in spinal cord tissues. (U) Immunofluorescence staining of SOD1 aggregates in the anterior horn motor neurons of spinal cords. (V) Quantification of motor neurons containing SOD1 aggregates. Data represent mean ± SD (*n* = 3). One‐way or two‐way ANOVA followed by Dunnett's post hoc test were used to evaluate statistical significance, *p < 0.05, **p < 0.01, ***p < 0.001, ****p < 0.0001, ns: not significant.

hSOD1^G93A^ transgenic ALS mice, which predominantly exhibit progressive muscle weakness and atrophy, are widely used as an animal model to study ALS mechanisms [6]. To evaluate the role of SIK2 in hSOD1^G93A^ transgenic mice, intraventricular injections of lentivirus‐SIK2‐shRNA were performed at 60 days of age. Four weeks post‐injection, immunofluorescence revealed high transfection efficiency in the ventral horn of the spinal cord (Figure 7A). The mRNA and protein levels of SIK2 were significantly reduced in LV‐shSIK2‐injected ALS mice compared to LV‐mcherry controls at 90 days of age (Figure 7B–D). The body weight of wild‐type (WT) mice steadily increased over time, while ALS mice exhibited a gradual decline with disease progression. LV‐shSIK2 treatment significantly mitigated weight loss in ALS mice compared to controls (Figure 7E). The rotarod test was utilized to assess motor function. WT mice consistently maintained the maximum hang duration of 180 s on the rotating rod throughout the study, whereas hSOD1^G93A^ mice maintained this duration only up to approximately 92 days of age. SIK2 down‐regulation alleviated the decline in motor function in hSOD1^G93A^ mice (Figure 7F). Furthermore, SIK2 suppression extended the average lifespan of ALS mice by 12 days and slightly postponed disease onset (Figure 7G,H). Loss of anterior horn motor neurons in the spinal cord is a hallmark of ALS pathology [53]. In this study, lumbar spinal cord tissue from 150‐day‐old ALS mice was analyzed using Nissl staining and immunohistochemistry. ALS mice exhibited a substantial reduction in the number of motor neurons in the anterior horn of the spinal cord compared to wild‐type mice. Consistent trends were observed in the results of both immunohistochemistry and Nissl staining. Notably, prolonged survival in LV‐shSIK2‐injected ALS mice was associated with reduced motor neuron loss in the ventral horn of the spinal cord compared to controls (Figure 7I–L). Additionally, HE staining of gastrocnemius muscle tissue from 150‐day‐old mice revealed that, compared to wild‐type (WT) mice, hSOD1^G93A^ mice exhibited highly irregular muscle fiber bundles, significantly enlarged inter‐fiber endomysial spaces, and aggregated nuclei, indicative of pronounced muscle atrophy. Notably, SIK2 inhibition significantly delayed neurogenic atrophy in hSOD1^G93A^ mice, consistent with the results of the rotarod test (Figure 7M).

To assess whether SIK2 down‐regulation alters the autophagy‐lysosome pathway in vivo, we performed Western blotting to evaluate LC3II and P62 protein levels. Consistent with our in vitro findings, LC3II and P62 levels were markedly increased in the spinal cords of ALS mice at the late disease stage. The LV‐shSIK2 treatment group exhibited a marked reduction in SIK2 expression in both wild‐type (WT) and ALS mice (Figure 7N,S). Notably, in LV‐shSIK2‐treated ALS mice, LC3II and P62 levels were significantly reduced compared to LV‐mCherry‐treated ALS mice, while no significant differences were observed between the two groups in WT mice (Figure 7N–P). Moreover, down‐regulation of SIK2 in hSOD1^G93A^ mice resulted in a notable increase in p‐mTOR (Ser2448) and p‐p70S6K (Thr389) levels (Figure 7N,Q,R). ALS mice treated with LV‐shSIK2 also showed a significant increase in mature CTSB levels (Figure 7N,T). To confirm the effect of SIK2 inhibition on SOD1 aggregates, immunofluorescence staining was conducted on 150‐day‐old mice. Compared to controls, the LV‐shSIK2‐injected group exhibited a reduced percentage of neurons containing SOD1 aggregates in the ventral horn of the spinal cord of ALS mice (Figure 7U,V). Taken together, these findings indicate that SIK2 down‐regulation prolongs the lifespan of ALS mice by enhancing autophagy‐mediated clearance of mutant SOD1 aggregates.

## 4 Discussion

ALS is a devastating neurodegenerative disease characterized by the progressive degeneration of motor neurons. Genetic studies have shown that mutations in autophagy‐related genes can contribute to ALS, highlighting the physiological role of the autophagic pathway in motor neuron degeneration [54]. SIK2 has been implicated in the regulation of the autophagic pathway [27, 28, 29, 55]. However, its role in ALS remains unexplored. In this study, we demonstrated that SIK2 expression levels were significantly increased in the motor neurons of both hSOD1^G93A^ transgenic mice and cellular models of ALS. Conversely, the knockdown of SIK2 expression reduced mutant SOD1 aggregation and enhanced neuronal survival in both in vivo and in vitro ALS models. Furthermore, SIK2 knockdown in the central nervous system delayed disease onset, improved motor function, and extended the lifespan of ALS mice. Our findings suggest that the neuroprotective effects of SIK2 inhibition are partially mediated by the activation of autophagic flux and mTORC1 signaling. Notably, we also showed that the overexpression of exogenous SIK2 impaired lysosomal acidification, resulting in defective autophagic flux and diminished mTORC1 activation, ultimately leading to motor neuron degeneration.

SIK2, a novel serine/threonine protein kinase and member of the AMP‐activated protein kinase family, is known to regulate multiple biological functions [26]. Notably, SIK2 is abundantly expressed in the central nervous system, and its knockdown has been shown to provide neuronal protection during ischemic injury [24]. Additionally, in microglia of mice with intracerebral hemorrhage, SIK2 inhibition reduced inflammation and ameliorated neurological dysfunction. Furthermore, SIK2 has been implicated in modulating hippocampal neurogenesis and exerting antidepressant‐like effects [23]. Despite these findings, the role of SIK2 in ALS has not been previously investigated. In this study, we report for the first time that SIK2 is up‐regulated in neurons in both cell culture and mouse models of ALS, suggesting its involvement in ALS progression. We hypothesize that the down‐regulation of SIK2 observed at earlier stages represents a compensatory response to mSOD1 aggregation. However, this endogenous suppression of SIK2 appears insufficient to sustain effective clearance of aggregates.

Increasing evidence suggests that elevated levels of autophagy markers and the accumulation of autophagosomes in the spinal cords of ALS patients and hSOD1^G93A^ mice indicate that autophagic alterations are associated with the pathogenesis of motor neuron degeneration [11, 15, 56, 57, 58]. Autophagy involves the formation of autophagosomes and their subsequent degradation in lysosomes. Effective autophagy flux requires a balance between the formation of autophagic vacuoles and their clearance by lysosomes. Interestingly, we observed a progressive increase in p62 levels in the spinal cords of ALS mice [59]. Since p62 is degraded along with aggregates in autolysosomes [60], these findings suggest the possibility of a blockade in autophagic flux, although p62 is also involved in proteasomal degradation [61]. Previous studies have shown that mutant SOD1 dysregulates autophagy flux [14, 62, 63, 64]. Specifically, mutant hSOD1^G93A^ interacts with the intermediate dynein chain, impairing retrograde transport and contributing to autophagy –lysosomal deficits [14]. Moreover, progressive lysosomal deficits have been observed during the asymptomatic stages in fALS‐linked hSOD1^G93A^ mice [14]. Consistent with previous findings, we detected significantly higher levels of LC3‐II in NSC34‐SOD1^G93A^ cells, correlating with autophagosomes accumulation. Notably, p62 levels were also elevated in NSC34‐hSOD1^G93A^ cells compared to NSC34‐SOD1^WT^, NSC34‐EV, and NSC34 cells. Remarkably, LC3‐II and p62 levels increased further after chloroquine treatment, indicating partial impairment of autophagic flux in NSC34‐SOD1^G93A^ cells. We speculate that this inhibition of autophagic flux may be related to lysosomal dysfunction. Further studies are required to investigate lysosomal function in cellular models of ALS. Collectively, our findings confirm that hSOD1^G93A^ causes a blockade in autophagic flux despite the induction of autophagic vacuoles formation.

SIK2 has been proposed to play a role in autophagy regulation [27, 28, 29, 45, 55]. It has been suggested that SIK2 activity is essential for autophagosome maturation and is post‐translationally regulated by p300/CBP and HDAC6 [27]. Alternatively, studies have shown that SIK2 can restrict autophagic flux [28] and regulate TFEB [29]. However, the mechanisms by which SIK2 regulates autophagy in ALS cell models remain unclear. Moreover, no prior connection between SIK2 and lysosomal function has been established. In this study, we demonstrated that SIK2 overexpression induces autophagy, consistent with previous findings showing that SIK2 represses the autophagic degradation of PHLPP2 and PP2A [45], and restricts autophagy in triple‐negative breast cancer [28]. We observed a substantial increase in the number of autophagosomes upon SIK2 upregulation. However, this increase was attributed to impaired lysosomal degradation. Functional lysosomes must maintain an acidic pH, which is critical for their degradative activity [65]. Here, we showed that SIK2 overexpression causes lysosomal alkalization, leading to diminished autophagic flux despite increased autophagy induction. Additionally, we found that SIK2 compromises mTORC1 activity. The mTOR complex 1 (mTORC1) is a major negative regulator of autophagy, as mTORC1‐mediated phosphorylation inhibits autophagosome formation by suppressing the Atg13‐ULK1/2 complex [49, 66]. It is likely that SIK2‐induced abrogation of mTORC1 signaling contributes to autophagy induction. Moreover, mTORC1 negatively regulates TFEB [67], a master regulator of lysosomal biogenesis. However, the increased autophagosomes observed were not associated with enhanced lysosomal activity, suggesting that SIK2 may regulate lysosomal function through an mTORC1‐independent mechanism. Recent studies indicate that lysosomes play critical roles in regulating mTORC1 signaling [19, 68]. Our findings suggest that SIK2 disrupts autophagic flux at the lysosomal degradation step, which may, in turn, inhibit mTORC1 activity. Active mTORC1 has anti‐apoptosis properties and protects neurons from oxidative stress [20, 69], implying that indiscriminate inhibition of mTORC1 could have detrimental effects under certain conditions. Notably, the mTORC1‐dependent autophagy pathway is activated in the spinal cords of ALS mice at 90 and 120 days of age [20]. In hSOD1^G93A^ mice, n‐BP has been shown to attenuate autophagic activity by enhancing mTOR signaling, providing neuroprotection [70]. Conversely, rapamycin treatment accelerates motor neuron degeneration in ALS models [17, 20], suggesting that mTORC1 activity is crucial for motor neuron survival. In contrast, in a frontotemporal dementia (FTD) transgenic mouse model, rapamycin treatment was neuroprotectivie [71]. The paradoxical results may be attributed to differences in disease models and the varying states of autophagic flux. Our findings indicate that SIK2 impairs mTORC1 activity and enhances autophagy induction. However, overexpression of SIK2 promotes defective autophagic flux, ultimately leading to motor neuron death. This finding aligns with a recent study that loss of ubiquitins compromises autophagy flux despite increased autophagy induction and eventually causes the demise of neurons [72]. Conversely, loss of SIK2 increases autophagy flux and promotes motor neuron survival. We propose that impaired autophagy flux in SIK2‐overexpressing cells may result from lysosomal dysfunction. Future studies should focus on elucidating the precise mechanisms by which SIK2 affects lysosomal function.

A hallmark of ALS is the presence of abnormal protein aggregates in degenerative motor neurons. While the role of these inclusions remains debated, it is generally accepted that misfolded proteins, including SOD1, are directly associated with neurotoxicity and motor dysfunction [73]. Previous studies have demonstrated that conformational changes induced by SOD1 mutations increase its propensity to aggregate [74]. Both large aggregates and soluble oligomers contribute to toxicity, at least in the context of SOD1‐mediated ALS [75]. Notably, recent findings suggest that the expression of mutant SOD1 in motor neurons is sufficient to cause MN death and locomotor deficits in ALS transgenic mice [73]. Thus, reducing the levels of disease‐related proteins presents a promising strategy for mitigating disease progression. Consistent with this concept, our current study demonstrates that SIK2 inhibition reduces mutant SOD1 misfolding and protects motor neurons from apoptosis. This effect is likely mediated by the activation of autophagic flux. It is well established that large protein aggregates are primarily degraded via the autophagy‐lysosomal pathway [76]. In our present study, we show that SIK2 inhibition enhances autophagic flux, accelerating the clearance of mutant SOD1 aggregates. Conversely, SIK2 overexpression not only increases the accumulation of mutant SOD1 protein aggregates but also sensitizes neurons to death by disrupting autophagic flux. In summary, we propose that the loss of SIK2 reduces mutant SOD1‐induced toxicity by lowering its expression levels and promoting its clearance through the autophagy‐lysosomal pathway.

Salt‐inducible kinase (SIK) was initially identified as a protein kinase in the adrenal cortex of rats, regulated by dietary high‐salt intake [77]. The SIK family consists of three isoforms, forming a subfamily of AMPK family kinases. It is widely accepted that SIKs are involved in maintaining metabolic homeostasis. Recent findings suggest that SIK family members also regulate neurons survival [24] and play critical roles in inflammatory responses [78, 79]. Notably, SIK1 has been associated with ALS‐specific copy number variations in a genome‐wide association study [80]. Moreover, in vitro screening of small molecule inhibitors has shown that the FDA‐approved drug bosutinib can induce an anti‐inflammatory phenotype in macrophages by targeting SIK2 [81, 82]. More importantly, bosutinib has been shown to increase the survival rate of iPSC‐derived motor neurons in ALS patients with SOD1 mutations [83], and it has been advanced to a phase I dose‐escalation clinical trial for ALS patients [84]. Our findings demonstrate that the loss of SIK2 rescues ALS‐related motor neuron degeneration and reduces the accumulation of misfolded mutant SOD1 protein. By focusing on motor neurons, we showed that SIK2 depletion enhances autophagy. However, the role of SIK2 in other cell types, such as microglia and astrocytes, was not explored in this study. Recent research has proposed combining microglial NF‐κB inhibition with mutant SOD1 aggregate clearance in motor neurons, resulting in additive improvements in lifespan and motor function [85]. In this context, it will be important for future studies to investigate the role of SIK2 in microglia and astrocytes to fully understand its contribution to ALS pathogenesis.

**FIGURE 8:**
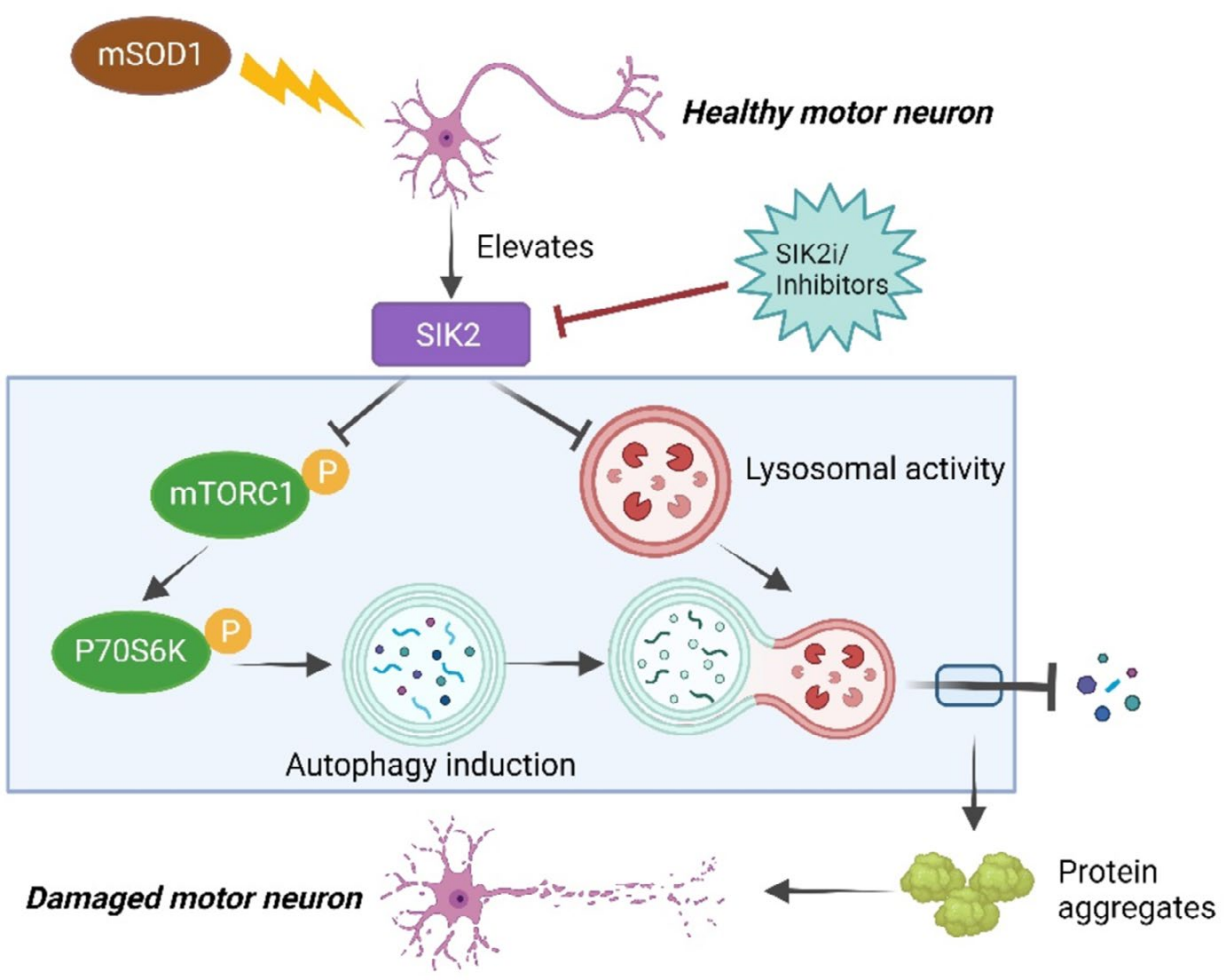
Schematic diagram of potential mechanisms by which SIK2 regulates autophagy in ALS. SIK2 contributes to the induction of autophagy by inhibiting mTORC1 signaling. However, SIK2 disrupts lysosomal dysfunction, ultimately resulting in defective autophagy flux and accumulation of mutant SOD1 aggregates. Inhibition of SIK2 promotes the removal of mutant SOD1 accumulation by activating autophagy flux and restores mTORC1 activity, thus playing a neuroprotective role on motor neurons in ALS.

In conclusion, our research demonstrates that SIK2 suppression exerts a neuroprotective effect in ALS cellular and animal models by activating autophagy‐mediated clearance of mutant SOD1 aggregates (Figure 8). These findings highlight SIK2 as a potential therapeutic target for the treatment of ALS.

## Author Contributions

Honglin Feng, Weiwei Liang, Chunting Zhang, and Di Wang designed the study. Weiwei Liang, Xiaoli Su, Xingli Tan, and Di Huo performed the cell experiments. Yueqing Yang, Chaohua Cong, Ying Wang, and Hongyong Wang performed animal experiments. Shuyu Wang and Xudong Wang helped analyze the data. Honglin Feng and Weiwei Liang participated in writing the manuscript. All authors have approved the final manuscript.

## Ethics Statement

All animal experiments were approved by the Animal Experiment Ethics Committee of Harbin Medical University. The ethical approval number is IACUC‐2022044.

## Conflicts of Interest

The authors declare no conflicts of interest.

## Supporting information


Data S1.

## Data Availability

All data included in the current study are available from the corresponding author upon reasonable request.
