# Supplementary figures and images for: Inhibition of Salt‐Inducible Kinase 2 Protects Motor Neurons From Degeneration in ALS by Activating Autophagic Flux and Enhancing mTORC1 Activity
Source: CNS Neurosci Ther. 2025 Mar 26;31(3):e70341. doi: 10.1111/cns.70341 (PMC11937914; doi:10.1111/cns.70341)

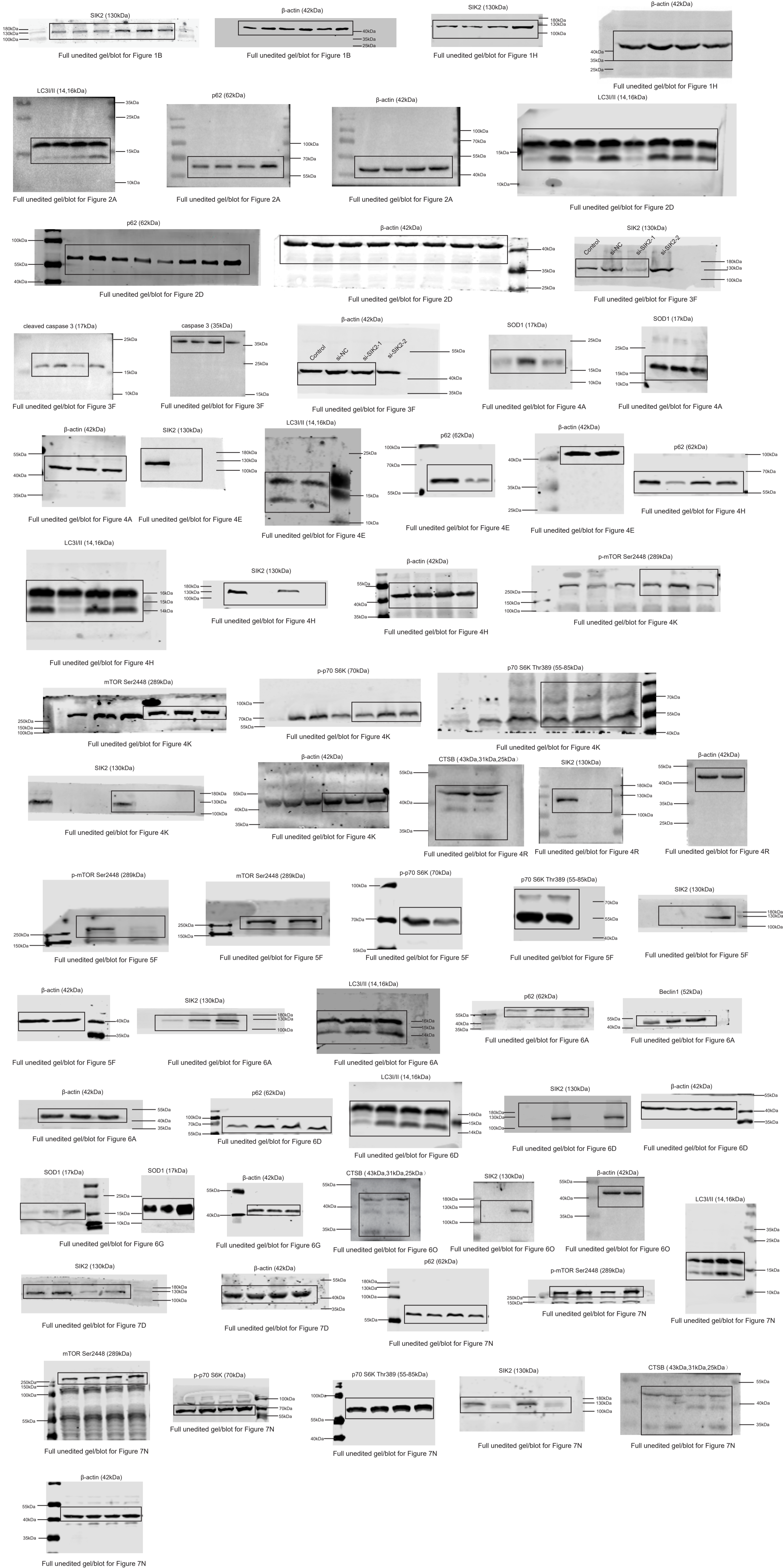

Supplement: Supplementary file 1 — Data S1. [file CNS-31-e70341-s001.pdf]
